# Sclerostin blockade inhibits bone resorption through PDGF receptor signaling in osteoblast lineage cells

**DOI:** 10.1172/jci.insight.176558

**Published:** 2024-05-07

**Authors:** Cyril Thouverey, Pierre Apostolides, Julia Brun, Joseph Caverzasio, Serge Ferrari

**Affiliations:** Service of Bone Diseases, Department of Medicine, University Hospital of Geneva, Geneva, Switzerland.

**Keywords:** Bone biology, Osteoclast/osteoblast biology, Osteoporosis, Signal transduction

## Abstract

While sclerostin-neutralizing antibodies (Scl-Abs) transiently stimulate bone formation by activating Wnt signaling in osteoblast lineage cells, they exert sustained inhibition of bone resorption, suggesting an alternate signaling pathway by which Scl-Abs control osteoclast activity. Since sclerostin can activate platelet-derived growth factor receptors (PDGFRs) in osteoblast lineage cells in vitro and PDGFR signaling in these cells induces bone resorption through M-CSF secretion, we hypothesized that the prolonged anticatabolic effect of Scl-Abs could result from PDGFR inhibition. We show here that inhibition of PDGFR signaling in osteoblast lineage cells is sufficient and necessary to mediate prolonged Scl-Ab effects on M-CSF secretion and osteoclast activity in mice. Indeed, sclerostin coactivates PDGFRs independently of Wnt/β-catenin signaling inhibition, by forming a ternary complex with LRP6 and PDGFRs in preosteoblasts. In turn, Scl-Ab prevents sclerostin-mediated coactivation of PDGFR signaling and consequent M-CSF upregulation in preosteoblast cultures, thereby inhibiting osteoclast activity in preosteoblast/osteoclast coculture assays. These results provide a potential mechanism explaining the dissociation between anabolic and antiresorptive effects of long-term Scl-Ab.

## Abstract 2

Sclerostin-targeting antibody exerts prolonged inhibition of bone resorption by preventing the formation of a sclerostin-LRP6-PDGFR ternary complex and the consequent coactivation of PDGFR signaling in preosteoblasts, independent of Wnt signaling.

## Graphical abstract

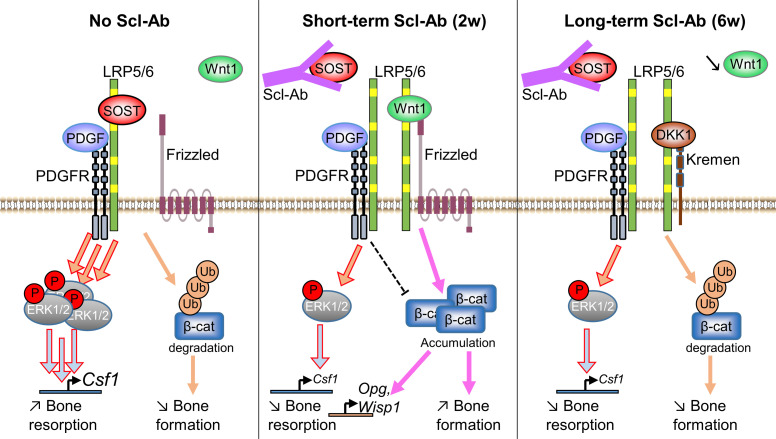

## Introduction

Sclerostin, encoded by the *SOST* gene, is an osteocyte-secreted protein that antagonizes low-density lipoprotein receptor–related proteins 5 and 6 (LRP5 and -6), thereby inhibiting canonical Wnt signaling in osteoblast lineage cells and bone formation (1–4). Owing to its restricted expression in the adult skeleton, sclerostin has emerged as an attractive therapeutic target to increase bone mass and strength in osteoporotic patients. Consequently, administration of antibodies targeting sclerostin (Scl-Abs) has been shown to augment bone mineral density and bone strength in humans, through transient elevation of bone formation and sustained reduction of bone resorption (5–10).

Mechanistically, short-term Scl-Ab treatment induces rapid and intense increases in serum procollagen type I N-terminal propeptide (PINP) and histomorphometric indices of bone formation, as well as a decrease in serum level of the bone resorption marker C-terminal telopeptides of type I collagen (CTX) in osteoporotic patients (7–10). Preclinical investigations have shown that Scl-Abs simultaneously activate modeling-based bone formation by stimulating transition of bone-lining cells into active osteoblasts and generate a positive bone balance at remodeling sites by enhancing the anabolic power (vigor) of each osteoblast (11–13). In parallel, bone resorption surfaces are decreased and associated with a lower receptor activator of NF-κB ligand (RANKL)/osteoprotegerin (OPG) ratio, decreased expression of *Csf1* (encoding macrophage colony–stimulating factor, M-CSF), essential for osteoclast differentiation and survival, and enhanced expression of *Wisp1*, a negative regulator of bone resorption (13, 14).

After 3 to 6 months of Scl-Ab treatment, serum PINP and bone formation indices return to initial values, whereas serum CTX and bone resorption parameters are maintained below baseline levels (7–10). Although overall bone turnover eventually decreases, the positive bone mineral balance within bone remodeling units is maintained, but attenuated, allowing significant bone mass gain to continue for the duration of therapy (1 year) (15). Counterregulation of bone formation with Scl-Ab administration can be explained by increased expression of Wnt pathway inhibitors such as *Sost* and *Dkk1* (encoding Dickkopf-related protein 1, DKK1), and a decreasing number of osteoprogenitors (14, 16). In this context, the reason why Scl-Abs exert prolonged anticatabolic effects despite attenuation of their bone anabolic activity remains unclear. A possible explanation is that sclerostin neutralization could reduce bone resorption independently of canonical Wnt signaling activation.

We previously showed that platelet-derived growth factor receptor α (PDGFRα) and PDGFRβ in *Osterix*-positive cells redundantly control osteoclastogenesis and bone resorption by upregulating expression of *Csf1* in mice (17), and that sclerostin can induce PDGFR signaling in osteoblast lineage cells in vitro (18). Therefore, we hypothesized that the prolonged anticatabolic effect of Scl-Ab treatment could result from an inhibition of PDGFR signaling independently of its Wnt-activating properties in osteoblast lineage cells.

## Results

### Scl-Ab transiently stimulates bone formation in both control and Pdgfr-cKO mice.

**Figure 1:**
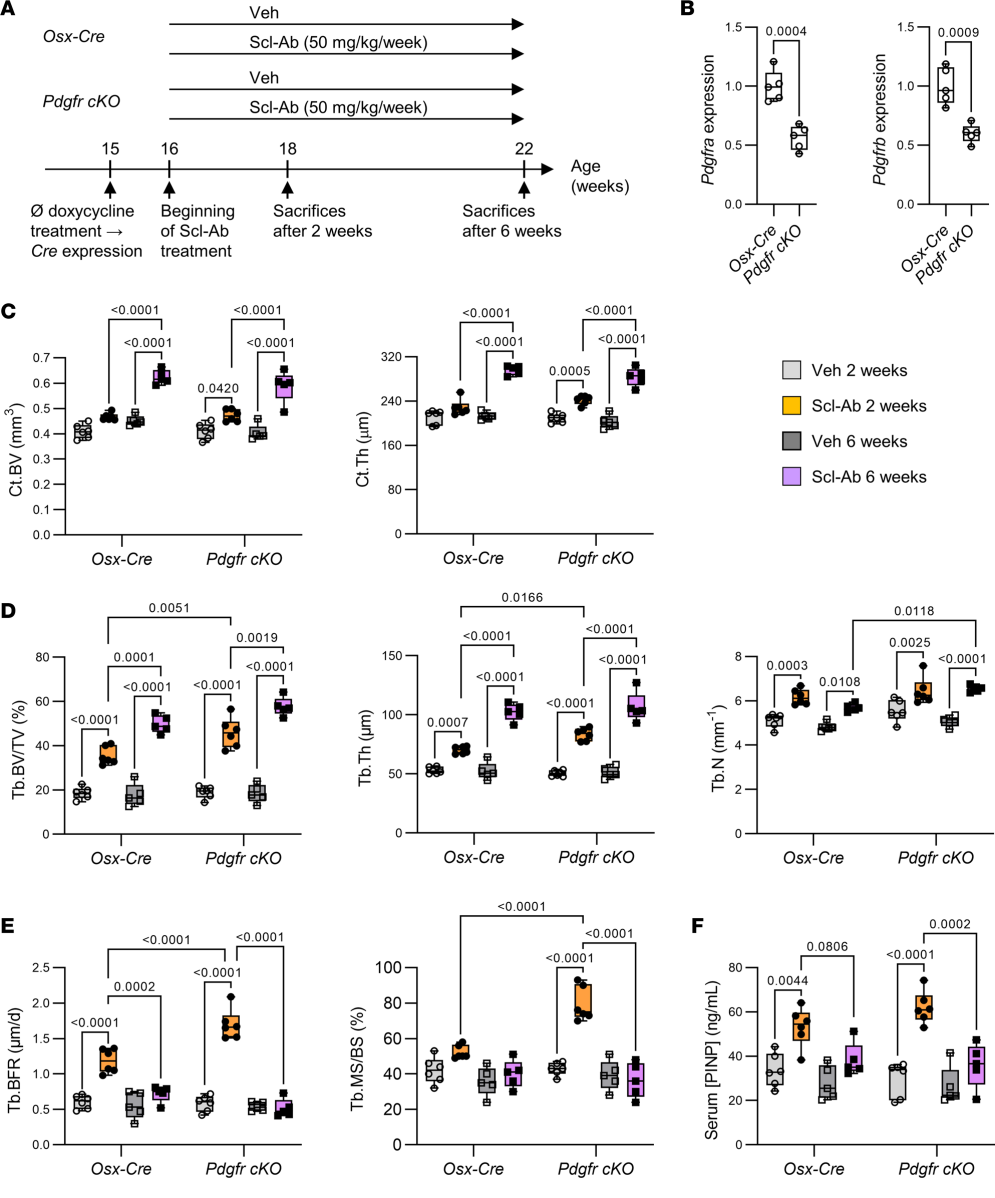
Scl-Ab–induced bone formation peaked more strongly in *Pdgfr-cKO* mice than in control mice after 2 weeks of treatment, but declined to baseline after 6 weeks of Scl-Ab treatment in mice of both genotypes. (**A**) Four-month-old *Osx-Cre* and *Pdgfr-cKO* (*Pdgfra cKO;Pdgfrb cKO*) male mice received subcutaneous injections of saline solution (Veh) or 25 mg/kg Scl-Ab twice a week for 2 or 6 weeks. *Cre* expression and/or conditional gene deletion was induced (by stopping doxycycline treatment) 1 week prior the beginning of Scl-Ab treatment. (**B**) Quantitative RT-PCR analyses of *Pdgfra* and *Pdgfrb* expression in bone marrow–free proximal tibial metaphyses at baseline (*n* = 5 per group). Differences between the 2 genotypes were analyzed using unpaired, 2-tailed *t* tests. (**C**) Cortical bone volume (Ct.BV) and thickness (Ct.Th) measured at tibial midshaft (*n* = 5–6 per group). (**D**) Trabecular bone microarchitecture measured at proximal tibiae (*n* = 5–6 per group). μCT parameters include bone volume/total volume (BV/TV), trabecular thickness (Tb.Th), and trabecular number (Tb.N). (**E**) Histomorphometric parameters of trabecular bone formation measured at the secondary spongiosa of distal femurs (*n* = 5–6 per group). Tb.BFR, trabecular bone formation rate; Tb.MS/BS, trabecular mineralizing surfaces/bone surfaces. (**F**) Serum levels of PINP (*n* = 5–6 per group). Data in **C**–**F** were analyzed by 3-way ANOVA followed by Tukey’s post hoc tests.

To test the role of PDGFR signaling in osteoblast lineage cells on Scl-Ab’s effects in vivo, we treated *Osx-Cre;Pdgfra^fl/fl^;Pdgfrb^fl/fl^* (hereafter *Pdgfr-cKO*) and control (*Osx-Cre*) mice with Scl-Ab or its vehicle solution (Veh) for 2 and 6 weeks (Figure 1A). Due to the short duration of PDGFR deletion (inducible Cre activation 1 week prior to treatment) (Figure 1B), *Pdgfr-cKO* and *Osx-Cre* mice treated with Veh showed similar cortical and trabecular bone mass at all time points (Figure 1, C and D). Scl-Ab increased cortical bone volume at the tibial midshaft, as well as trabecular number, thickness, and bone volume at the proximal metaphysis at 2 and 6 weeks in both genotypes (Figure 1, C and D, and Supplemental Figure 1A; supplemental material available online with this article; https://doi.org/10.1172/jci.insight.176558DS1). However, Scl-Ab increased trabecular bone volume more in *Pdgfr-cKO* mice than in *Osx-Cre* mice after 2 weeks, a trend that persisted after 6 weeks of treatment (Figure 1D and Supplemental Figure 1A). Scl-Ab treatment initially stimulated the mineral apposition rate on trabecular bone surfaces equally in both genotypes (i.e., at 2 weeks), but increased trabecular mineralizing surfaces and bone formation rate more in *Pdgfr-cKO* mice than in *Osx-Cre* mice (Figure 1E and Supplemental Figure 1, B and C). Bone formation parameters returned to pretreatment levels after 6 weeks of Scl-Ab treatment in mice of both genotypes (Figure 1E and Supplemental Figure 1, B and C). Similarly, serum levels of PINP tended to peak at a higher level in *Pdgfr-cKO* mice than in *Osx-Cre* mice after 2 weeks of Scl-Ab treatment, but declined to similar levels in both genotypes after 6 weeks of treatment (Figure 1F).

These observations indicated that PDGFR signaling could attenuate the extent of bone-forming surfaces in response to Scl-Ab, but did not play a role in the counterregulation of Scl-Ab’s effects on bone formation.

### Self-regulation of Wnt signaling in response to Scl-Ab is independent of PDGFRs.

**Figure 2:**
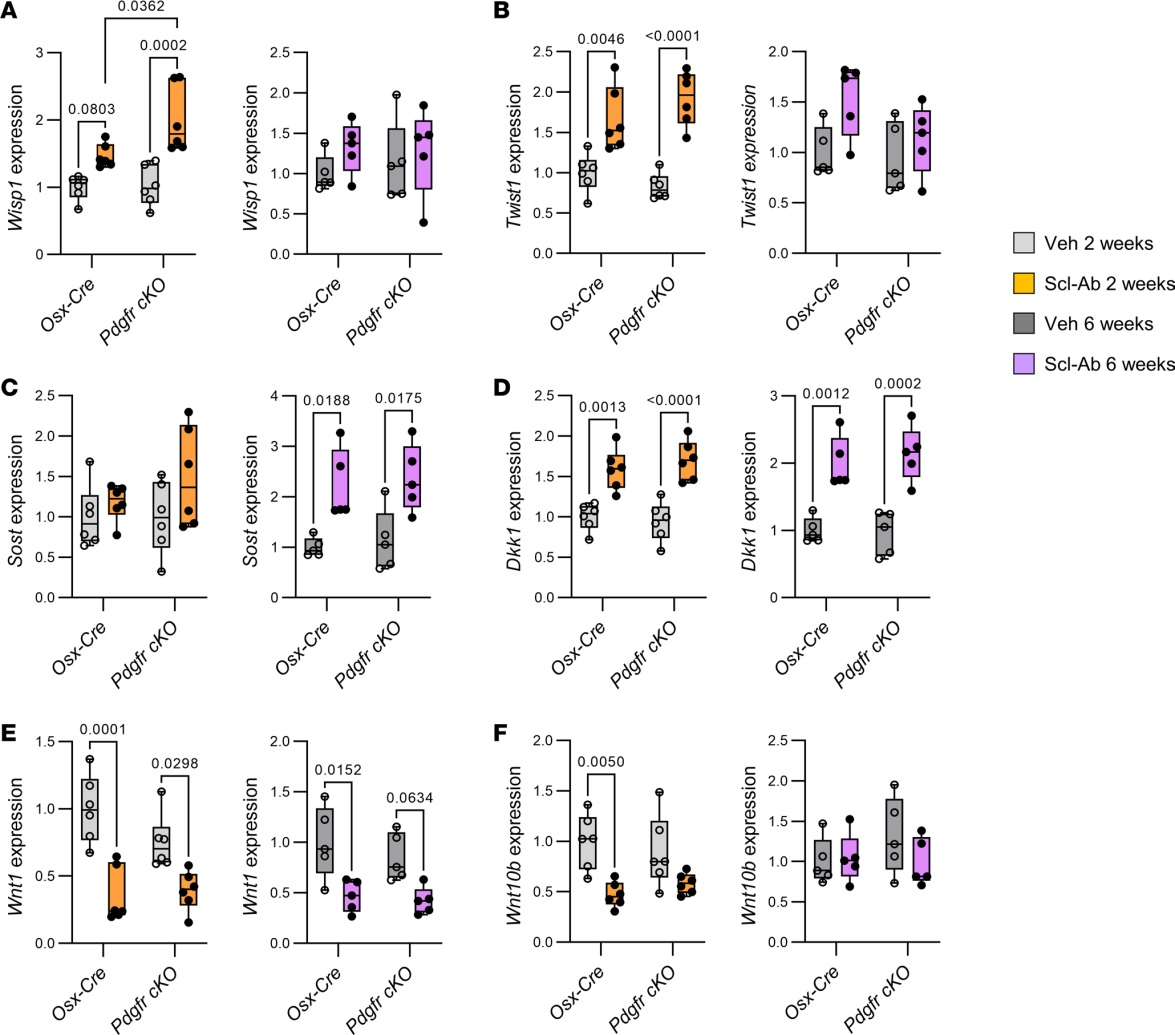
Self-regulation of Scl-Ab–induced Wnt signaling in bone coincided with increased expression of sclerostin and decreased expression of Wnt1 class of proteins in mice of both genotypes. Four-month-old *Osx-Cre* and *Pdgfr-cKO* male mice received subcutaneous injections of saline solution (Veh) or 25 mg/kg Scl-Ab twice a week for 2 weeks or 6 weeks. *Cre* expression and/or conditional gene deletion were induced 1 week prior the onset of Scl-Ab treatment. (**A**–**F**) Quantitative RT-PCR analyses of (**A**) *Wisp1* (encoding Wnt1-inducible-signaling pathway protein 1), (**B**) *Twist1* (twist-related protein 1), (**C**) *Sost* (sclerostin), (**D**) *Dkk1* (Dickkopf-related protein 1), (**E**) *Wnt1*, and (**F**) *Wnt10b* expression in proximal tibial metaphyses (*n* = 5–6 per group). Data were analyzed by 2-way ANOVA followed by Tukey’s post hoc test.

The similar decrease in Scl-Ab’s anabolic effects after 6 weeks in both *Pdgfr-cKO* and control mice suggested that prolonged Scl-Ab treatment is associated with a self-regulation of Wnt signaling that is independent of PDGFR signaling. Hence, we measured expression of selected Wnt target genes and Wnt pathway regulators in proximal tibial metaphysis isolated from *Osx-Cre* and *Pdgfr-cKO* mice. Two-week Scl-Ab treatment enhanced expression of *Wisp1* and *Twist1*, 2 Wnt target genes that are responsive to sclerostin neutralization (13, 14), even more strongly in *Pdgfr-cKO* mice than in *Osx-Cre* mice (Figure 2, A and B). Expression of both Wnt target genes returned to basal levels following 6 weeks of Scl-Ab treatment in both genotypes, thereby confirming attenuation of Wnt signaling with prolonged exposure to Scl-Ab (Figure 2, A and B). This downregulation of Wnt target gene expression was associated with a significant increase in *Sost* (after 6 weeks) and *Dkk1* (from 2 weeks) expression in both genotypes (Figure 2, C and D). Moreover, Scl-Ab administration quickly reduced expression of *Wnt1* and *Wnt10b* in bones of mice from both genotypes (Figure 2, E and F).

Altogether, those results indicated that Wnt signaling attenuation following prolonged Scl-Ab treatment was mediated by a negative feedback mechanism involving increased expression of Wnt signaling inhibitors and decreased expression of Wnt1 class of ligands, independently of PDGFR signaling.

### PDGFRs mediate Scl-Ab’s inhibitory effects on bone resorption and M-CSF secretion.

**Figure 3:**
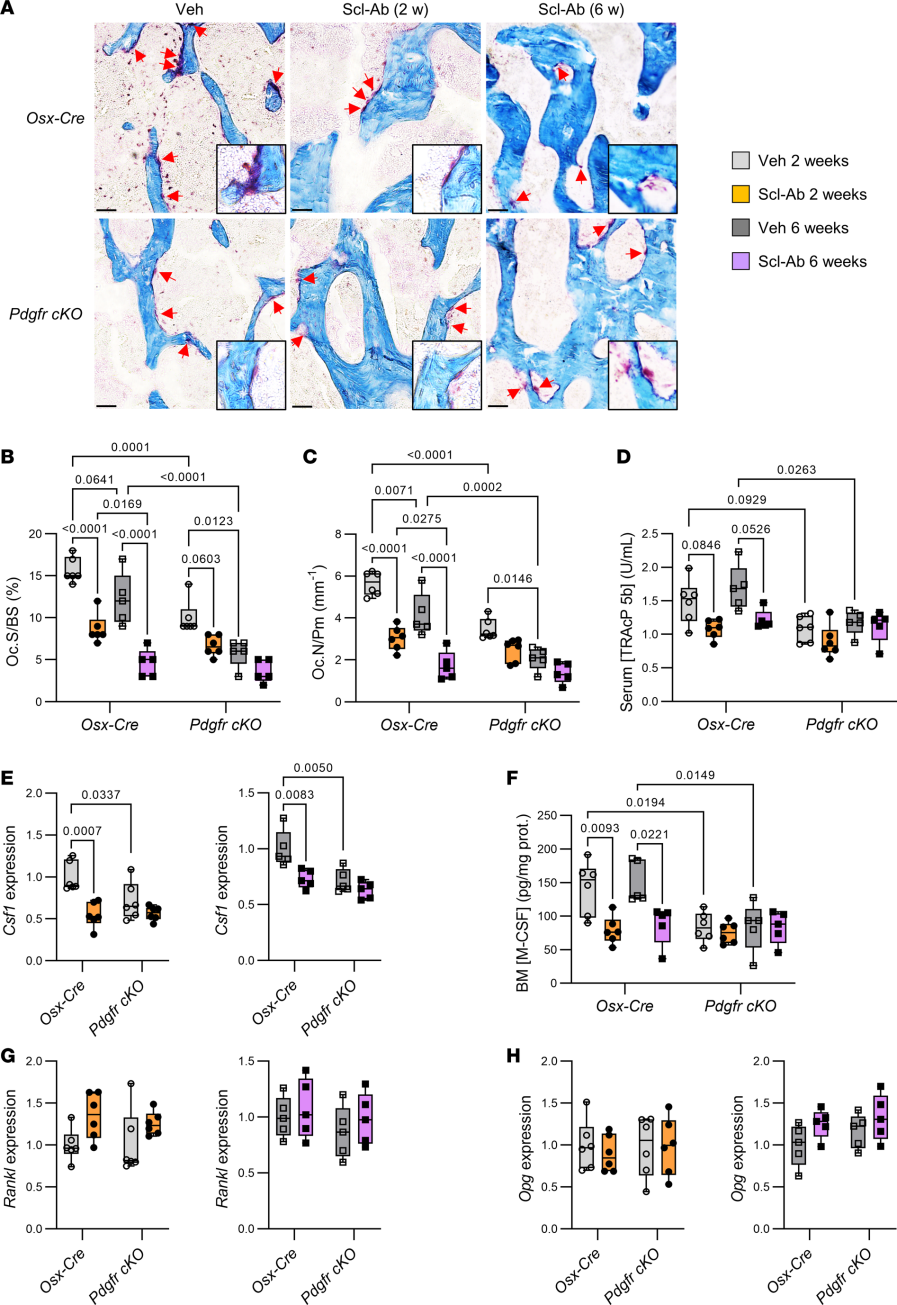
Scl-Ab treatment durably decreased bone resorption and *Csf1* expression in control mice, but did not exert any further anticatabolic effect in *Pdgfr-cKO* mice. Four-month-old *Osx-Cre* and *Pdgfr-cKO* (*Pdgfra cKO;Pdgfrb cKO*) male mice received subcutaneous injections of saline solution (Veh) or 25 mg/kg Scl-Ab twice a week for 2 or 6 weeks. *Cre* expression and/or conditional gene deletion were induced (by stopping doxycycline treatment) 1 week prior the beginning of Scl-Ab treatment. (**A**) Representative images of TRAP-stained histological sections of distal femurs. Scale bars: 50 μm. Original magnification, ×200 (high-magnification insets). (**B** and **C**) Histomorphometric parameters of trabecular bone resorption measured at the secondary spongiosa of distal femurs (*n* = 5–6 per group). Oc.S/BS, osteoclast surface/bone surface; Oc.N/Pm, osteoclast number/bone perimeter. (**D**) Serum levels of TRAcP 5b (*n* = 5–6 per group). (**E**) Quantitative RT-PCR analyses of *Csf1* (encoding M-CSF) expression in proximal tibial metaphyses (*n* = 5–6 per group). (**F**) M-CSF protein levels in bone marrow (BM) supernatants (*n* = 5–6 per group). (**G** and **H**) Quantitative RT-PCR analyses of (**G**) *Rankl* (receptor activator of NF-κB ligand) and (**H**) *Opg* (osteoprotegerin) expression in proximal tibial metaphyses (*n* = 5–6 per group). Data in **B**–**D** and **F** were analyzed by 3-way ANOVA followed by Tukey’s post hoc test. Data in **E**, **G**, and **H** were analyzed by 2-way ANOVA followed by Tukey’s post hoc test.

The suppression of PDGFRs in osteoblast lineage cells significantly decreased osteoclast number and surfaces and tended to reduce serum tartrate-resistant acid phosphatase isoform 5b (TRAcP 5b) levels (Figure 3, A–D), together with reduced *Csf1* expression and M-CSF protein levels in the bone microenvironment (Figure 3, E and F). Scl-Ab significantly reduced osteoclast number and surfaces in control mice after 2 and 6 weeks (Figure 3, A–C), while it tended to further diminish the already low osteoclast number and surfaces in *Pdgfr-cKO* mice after 2 weeks, but not after 6 weeks of treatment (Figure 3, A–C). Scl-Ab also tended to decrease serum levels of TRAcP 5b in *Osx-Cre* mice, but not in *Pdgfr-cKO* mice (Figure 3D). Accordingly, sclerostin neutralization lowered *Csf1* expression in bone and M-CSF protein levels in the bone marrow of control *Osx-Cre* mice but not in those of *Pdgfr-cKO* mice after 2 and 6 weeks of treatment (Figure 3, E and F). In contrast, we found no significant differences in *Rankl* or *Opg* expression in response to Scl-Ab treatment and PDGFR deletion (Figure 3, G and H).

Those data indicated that sclerostin and PDGFRs operated within the same signaling pathway to durably inhibit M-CSF secretion and bone resorption.

### Scl-Ab inhibits expression of PDGFR-responsive genes in bone without downregulating PDGFR expression.

**Figure 4:**
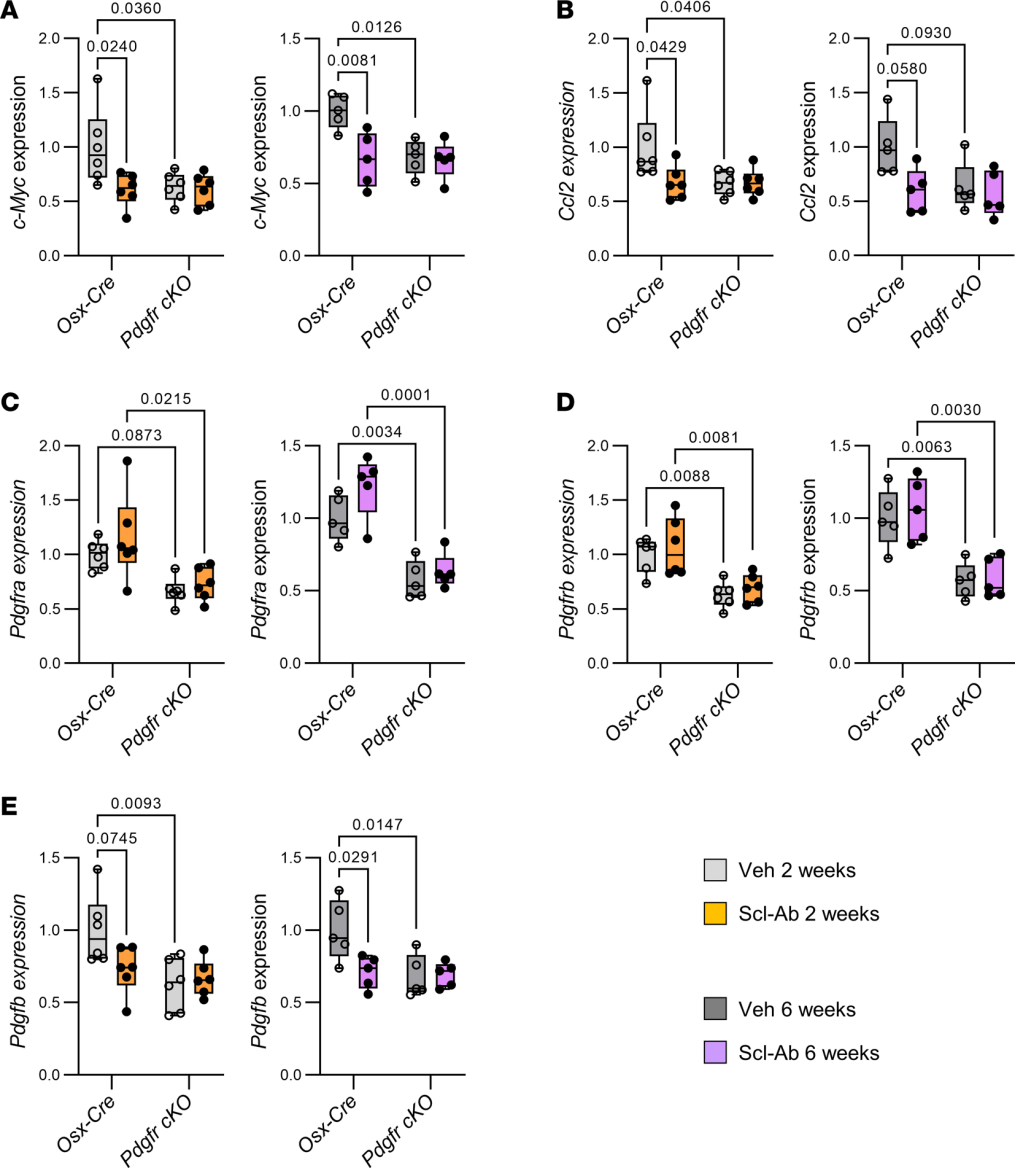
Scl-Ab treatment durably decreased expression of PDGFR target genes in bone of control mice, but did not exert any further inhibitory effect in *Pdgfr-cKO* mice. Four-month-old *Osx-Cre* and *Pdgfr-cKO* male mice received subcutaneous injections of saline solution (Veh) or 25 mg/kg Scl-Ab twice a week for 2 weeks or 6 weeks. *Cre* expression and/or conditional gene deletion were induced 1 week prior the onset of Scl-Ab treatment. (**A**–**E**) Quantitative RT-PCR analyses of (**A**) *c-Myc* (encoding myc pro-oncogenic protein), (**B**) *Ccl2* (C-C motif chemokine 2), (**C**) *Pdgfra* (PDGFRα), (**D**) *Pdgfrb* (PDGFRβ), and (**E**) *Pdgfb* (PDGF-B) expression in proximal tibial metaphyses (*n* = 5–6 per group). Data were analyzed by 2-way ANOVA followed by Tukey’s post hoc test.

To confirm that Scl-Ab–mediated inhibition of bone resorption is associated with a concomitant inhibition of PDGFR signaling, we measured expression of PDGFR target genes and PDGFR signaling components in proximal tibial metaphysis isolated from *Osx-Cre* and *Pdgfr-cKO* mice treated with Veh and Scl-Ab for 2 and 6 weeks. Selective suppression of *Pdgfra* and *Pdgfrb* in osteoblast lineage cells was associated with reduced expression of *c-Myc* and *Ccl2* (Figure 4, A and B), 2 PDGFR target genes (19, 20), and confirmed by reduced expression of both genes in *Pdgfr-cKO* mice (Figure 4, C and D). In contrast with the transient effect of Scl-Ab on Wnt target gene expression (Figure 2), Scl-Ab treatment consistently decreased expression of *c-Myc* and *Ccl2* in *Osx-Cre* mice after 2 and 6 weeks, but did not further reduce the already low expression levels of these genes in *Pdgfr-cKO* mice (Figure 4, A and B). In contrast, Scl-Ab did not affect *Pdgfra* and *Pdgfrb* expression levels (Figure 4, C and D). Furthermore, both selective PDGFR ablation in osteoblast lineage cells and Scl-Ab administration reduced expression of *Pdgfb*, encoding the PDGF-B ligand (Figure 4E), probably reflecting a low number of *Pdgfb*-expressing osteoclasts under these conditions (17).

These data indicated that Scl-Ab directly inhibited the expression of PDGFR signaling target genes and these effects were not counterregulated by the increased expression of Wnt inhibitors.

### Sclerostin potentiates PDGF-BB–stimulated Csf1 expression and bone resorption in vitro by forming a sclerostin-LRP6-PDGFR ternary complex for ERK1/2 activation.

**Figure 5:**
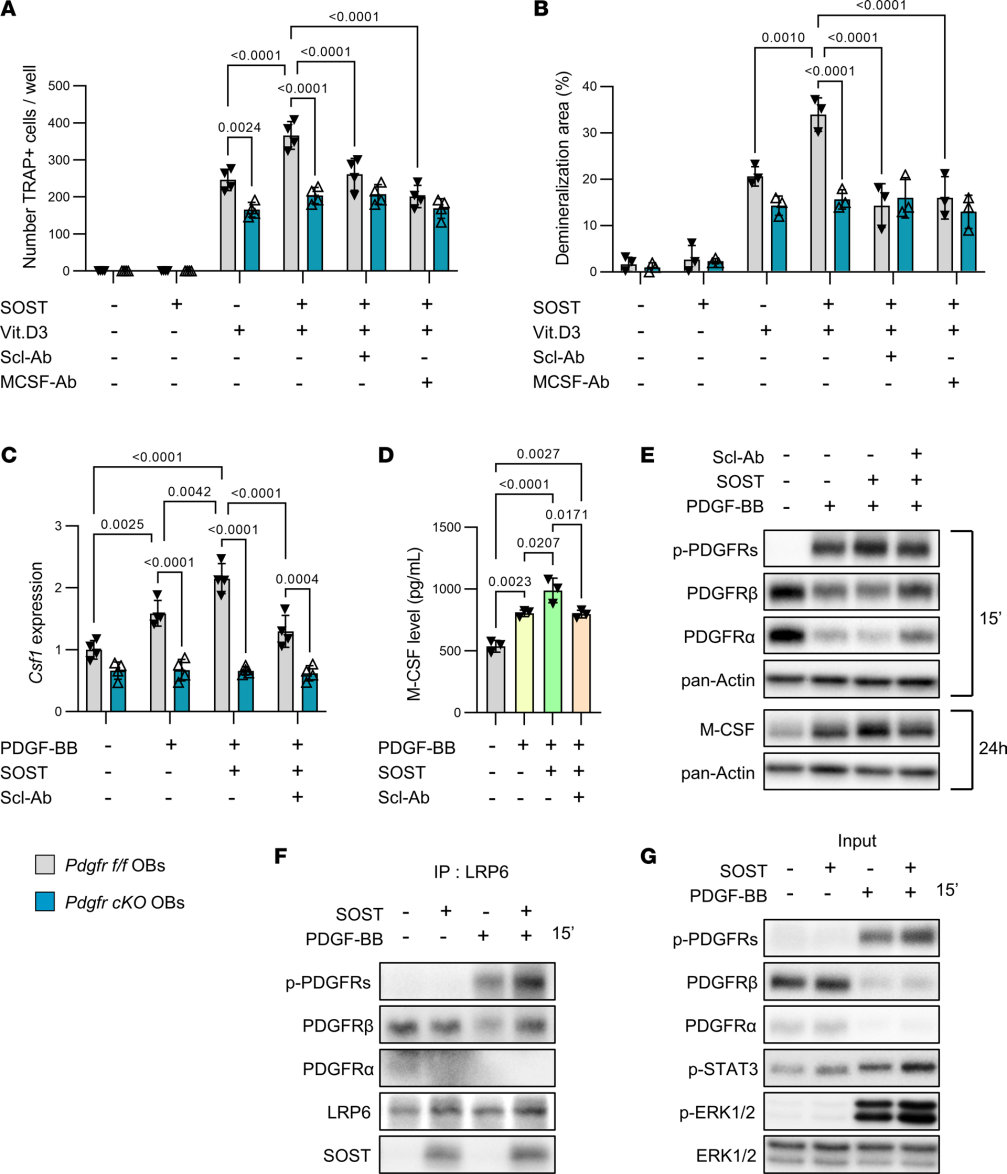
Scl-Ab blocked sclerostin-mediated PDGFR coactivation and M-CSF secretion in preosteoblast cultures, and calcitriol-induced osteoclast formation and activity in cocultures. (**A**) *Pdgfr^fl/fl^* (control) and *Pdgfr-cKO* (without PDGFRs) preosteoblasts were cocultured with nonadherent bone marrow cells isolated from WT mice in the presence of Veh, 10^–8^ M 1,25-dihydroxyvitamin D3 (calcitriol, Vit.D3), and/or 250 ng/mL recombinant sclerostin (SOST), with or without 1.25 μg/mL Scl-Ab or 500 ng/mL anti–M-CSF antibody (MCSF-Ab) for 8 days before quantification of TRAP-positive multinucleated cells. (**B**) The same cocultures performed in synthetic matrix–coated multiwell plates for 15 days before quantification of demineralized areas. (**C**) *Pdgfr^fl/fl^* and *Pdgfr-cKO* preosteoblasts were pretreated with Veh, 250 ng/mL SOST with and without 1.25 μg/mL Scl-Ab for 1 hour, and treated with Veh or 25 ng/mL PDGF-BB for 24 hours before measurement of *Csf1* expression by quantitative RT-PCR. Data in **A**–**C** were analyzed by 2-way ANOVA followed by Tukey’s post hoc test. (**D**) WT preosteoblasts were pretreated with Veh, 250 ng/mL SOST with and without 1.25 μg/mL Scl-Ab for 1 hour, and treated with Veh or 25 ng/mL PDGF-BB for 48 hours before quantification of M-CSF in culture media by ELISA. Data in **D** were analyzed by 1-way ANOVA followed by Tukey’s post hoc test. (**E**) WT preosteoblasts were pretreated with Veh or 500 ng/mL SOST with and without 2.5 μg/mL Scl-Ab for 1 hour, and then treated with 15 ng/mL PDGF-BB for the indicated time periods. PDGFR signaling and M-CSF protein level were determined by Western blot analyses. (**F** and **G**) WT preosteoblasts were pretreated with Veh or 500 ng/mL SOST for 2 hours, and then treated with 25 ng/mL PDGF-BB for 15 minutes. (**F**) Proteins were immunoprecipitated by anti-LRP6 antibody and detected by Western blot analyses. (**G**) PDGFR signaling was assessed in the remaining cell lysates.

To confirm and expand these in vivo observations, we analyzed the molecular mechanisms by which sclerostin could interfere with PDGFR signaling to regulate osteoclast development and function in vitro. Since PDGFRα and PDGFRβ expression was high in early osteoblasts expressing *Runx2* and *Col1a1*, and then declined as those cells differentiated into mature osteoblasts expressing *Ocn*, we used primary cultures of preosteoblasts (Supplemental Figure 2). Recombinant sclerostin did not have any effect on in vitro osteoclastogenesis in basal conditions, but enhanced calcitriol-induced osteoclastogenesis and demineralization of a synthetic matrix in preosteoblast/osteoclast cocultures (Figure 5, A and B, and Supplemental Figure 3, A and B). These effects were blocked by Scl-Ab, M-CSF–targeting antibody, or by PDGFR deletion in preosteoblasts (Figure 5, A and B, and Supplemental Figure 3, A and B). In addition, sclerostin potentiated PDGF-BB–induced *Csf1* expression and M-CSF secretion in preosteoblast cultures (Figure 5, C and D). Again, those effects were blocked by either Scl-Ab treatment (Figure 5, C and D) or suppression of PGDFRs in preosteoblasts (Figure 5C). It should be noted that sclerostin, Scl-Ab, or PDGFR deletion did not alter osteoblast differentiation during those cell culture experiments (Supplemental Figure 3, B and C). Consistent with those findings, sclerostin amplified PDGF-BB–induced phosphorylation of PDGFRs in preosteoblast cultures after 15 minutes and elevation of M-CSF protein level after 24 hours, effects that were abrogated in the presence of Scl-Ab (Figure 5E). Eventually, immunoprecipitation of proteins by anti-LRP6 antibody showed that sclerostin could intensify PDGF-BB–induced activation of PDGFRs by forming a ternary complex with LRP6 and PDGFRs in preosteoblasts (Figure 5F). Sclerostin-mediated potentiating effects on PDGF-BB–dependent PDGFR activation resulted in further stimulation of downstream PDGFR signaling pathways such as ERK1/2 and STAT3 in preosteoblasts (Figure 5G).

### Sclerostin potentiates PDGF-BB–induced Csf1 expression independently of Wnt/β-catenin signaling in preosteoblast cultures.

**Figure 6:**
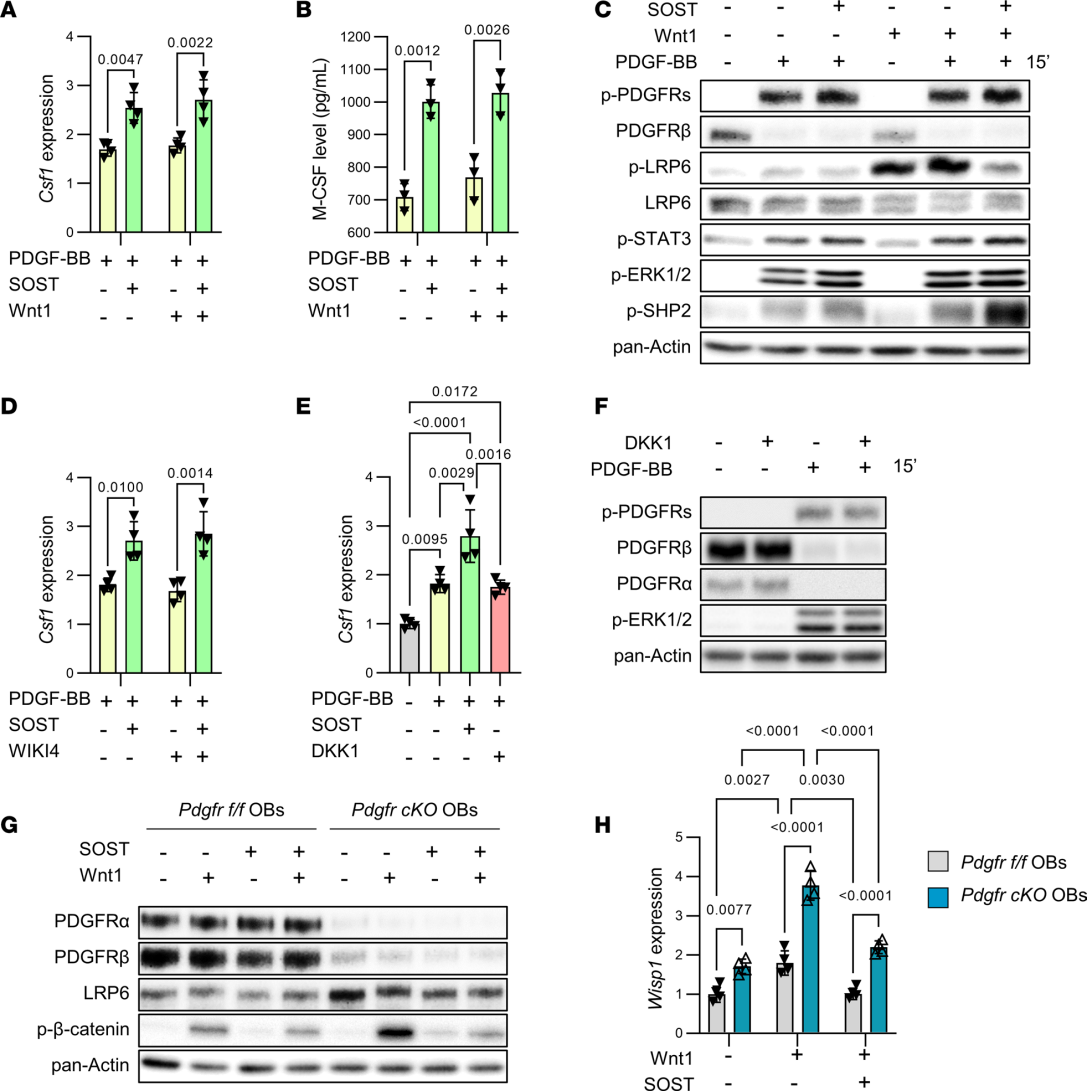
Sclerostin increases *Csf1* expression independently of Wnt/β-catenin signaling inhibition in preosteoblast cultures. (**A** and **B**) WT preosteoblasts were pretreated with Veh or 100 ng/mL Wnt1 with and without 500 ng/mL SOST for 2 hours, and treated with Veh or 25 ng/mL PDGF-BB for (**A**) 24 hours before measurement of *Csf1* expression by quantitative RT-PCR, or (**B**) 48 hours before quantification of M-CSF in culture media by ELISA. Data in **A** and **B** were analyzed by 2-way ANOVA followed by Tukey’s post hoc test. (**C**) WT preosteoblasts were pretreated with Veh or 100 ng/mL Wnt1 with and without 500 ng/mL SOST for 2 hours, and treated with Veh or 25 ng/mL PDGF-BB for 15 minutes before evaluation of PDGFR signaling by Western blot analyses. (**D**) WT preosteoblasts were pretreated with DMSO or 5 μM WIKI4 (inhibitor of Wnt/β-catenin signaling), and Veh or 500 ng/mL SOST for 2 hours, and treated with Veh or 25 ng/mL PDGF-BB for 24 hours before measurement of *Csf1* expression by quantitative RT-PCR. Data in **D** were analyzed by 2-way ANOVA followed by Tukey’s post hoc test. (**E**) WT preosteoblasts were pretreated with Veh, 500 ng/mL SOST, or 500 ng/mL DKK1 for 2 hours, and then treated with Veh or 25 ng/mL PDGF-BB for 24 hours before measurement of *Csf1* expression by quantitative RT-PCR. Data in **E** were analyzed by 1-way ANOVA followed by Tukey’s post hoc test. (**F**) WT preosteoblasts were pretreated with Veh or 500 ng/mL DKK1 for 2 hours, and then treated with Veh or 25 ng/mL PDGF-BB for 15 minutes before evaluation of PDGFR signaling by Western blot analyses. (**G**) *Pdgfr^fl/fl^* and *Pdgfr-cKO* preosteoblasts were pretreated with Veh or 500 ng/mL SOST, and then treated with Veh or 100 ng/mL Wnt1 for 2 hours before determination of Wnt/β-catenin signaling by Western blot analyses. (**H**) *Pdgfr^fl/fl^* and *Pdgfr-cKO* preosteoblasts were pretreated with Veh or 500 ng/mL SOST, and then treated with Veh or 100 ng/mL Wnt1 for 24 hours before measurement of *Wisp1* expression by quantitative RT-PCR. Data in **H** were analyzed by 2-way ANOVA followed by Tukey’s post hoc test.

To provide an explanation for the continuous inhibition of PDGFR signaling and *Csf1* expression despite self-attenuation of Wnt signaling in response to prolonged Scl-Ab exposure (Figure 4), we tested whether Wnt/β-catenin signaling activation or inhibition could influence sclerostin’s ability to coactivate PDGFRs and stimulate *Csf1* expression in preosteoblast cultures. First, the presence of Wnt1 in culture medium did not alter the potentiating effects of sclerostin on PDGF-BB–induced *Csf1* expression, M-CSF production, and PDGFR signaling activation (Figure 6, A–C). Second, inhibition of β-catenin signaling by WIKI4 had no effect on PDGF-BB–dependent sclerostin-mediated *Csf1* expression (Figure 6D). Third, in contrast with sclerostin, another Wnt signaling inhibitor, DKK1, could not potentiate PDGF-BB–mediated *Csf1* expression and PDGFR/ERK1/2 activation (Figure 6, E and F). Together, those results indicated that sclerostin could potentiate PDGF-BB–mediated *Csf1* expression and PDGFR activation independently of Wnt/β-catenin signaling inhibition. As a control, sclerostin’s capability to inhibit Wnt1-induced β-catenin activation and *Wisp1* expression was confirmed in preosteoblast cultures (Figure 6, G and H). In this context, it is noteworthy that suppression of PDGFRs in preosteoblasts could potentiate Wnt1-promoted β-catenin activation and *Wisp1* expression (Figure 6, G and H), thereby also explaining the potentiating effect of osteoblast lineage–selective ablation of PDGFRs on Scl-Ab–stimulated bone formation (Figure 1).

## Discussion

Sclerostin blockade exerts dual effects on bone, resulting in a potent but transient stimulation of bone formation, but a milder and sustained inhibition of bone resorption. Because Scl-Ab’s effects on bone formation are primarily mediated by stimulation of the Wnt/β-catenin signaling pathway in osteoblast lineage cells and this pathway is rapidly downregulated by the overexpression of Wnt inhibitors, including sclerostin itself and DKK1 (14, 16), the persistent inhibition of bone resorption suggests that sclerostin and its pharmacological inhibitors control osteoclastogenesis by an alternative, Wnt-independent pathway, in osteoblast lineage cells. Consistent with human data, we showed that, despite continuous cortical and trabecular bone mass gain, bone formation induced by Scl-Ab treatment in mice was transient. The decrease in Scl-Ab’s osteoanabolic effects was associated with attenuation of Wnt signaling due to elevated expression of Wnt signaling inhibitors, and decreased expressions of Wnt1 class of ligands. In contrast, bone PDGFR signaling, *Csf1* expression, M-CSF secretion, and bone resorption were durably reduced by Scl-Abs in control mice. *Pdgfr-cKO* mice recapitulated these changes and, although short-term Scl-Ab treatment tended to further decrease bone resorption in these mice, prolonged Scl-Ab exposure did not. Scl-Ab abolished sclerostin-mediated coactivation of PDGFR signaling and consequent M-CSF upregulation in preosteoblast cultures, and osteoclast formation and activity in preosteoblast/osteoclast coculture assays. Eventually, we showed that sclerostin could potentiate PDGFR activation, unlike DKK1 and independently of the presence of Wnt ligand, by forming a ternary complex with LRP6 and PDGFRs in preosteoblasts.

**Figure 7:**
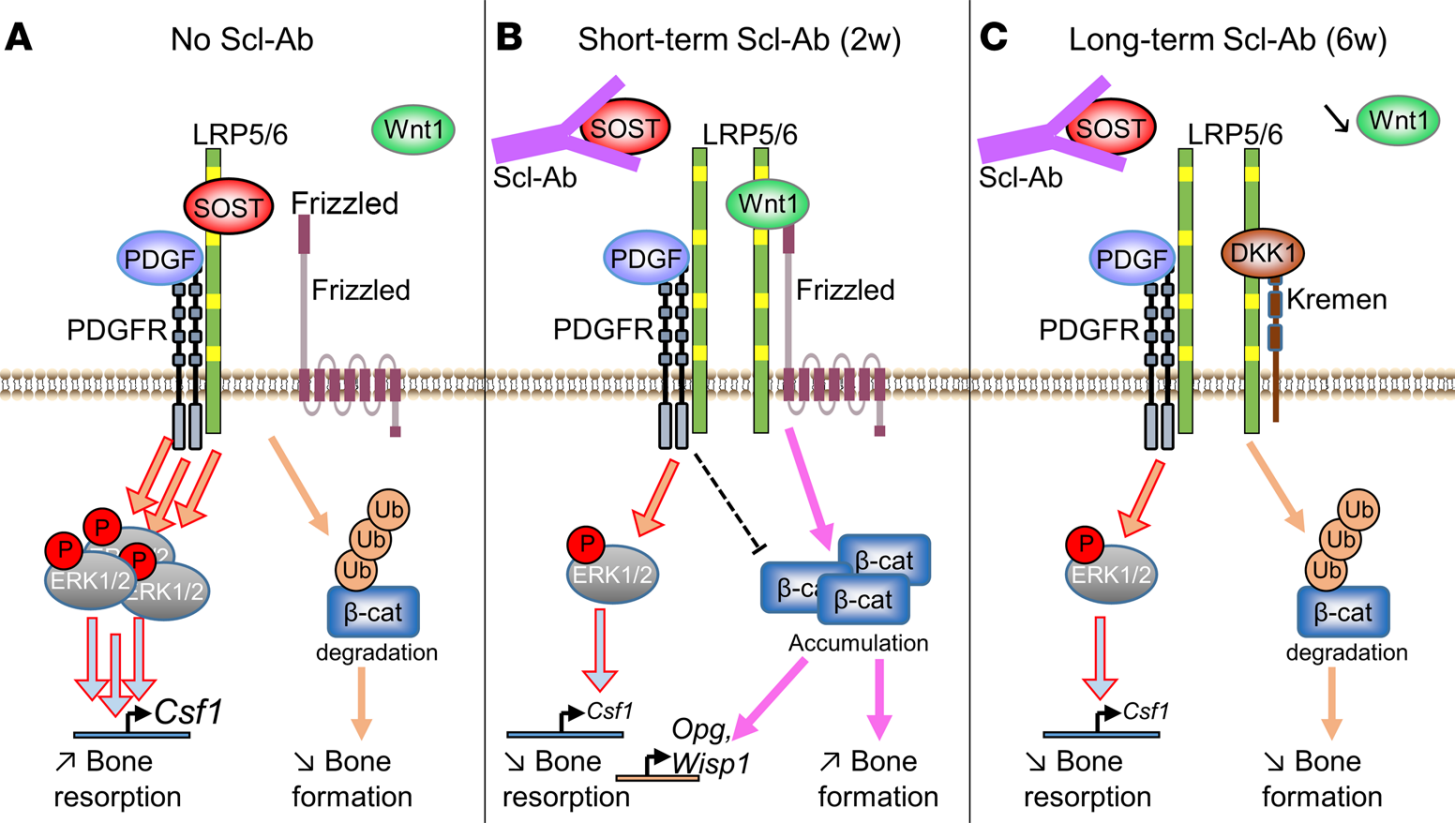
Proposed molecular mechanisms for Scl-Ab actions. (**A**) Besides its function as a Wnt/LRP6 antagonist that promotes β-catenin degradation by the proteasome, sclerostin forms a ternary complex with LRP6 and PDGFRs, leading to coactivation of PDGF-BB/PDGFR/ERK1/2 signaling and *Csf1* expression. (**B**) Short-term Scl-Ab exposure (2 weeks) prevents sclerostin binding to LRP6, thereby promoting Wnt1 class–induced β-catenin accumulation and signaling, and preventing sclerostin-mediated coactivation of PDGFR/ERK1/2 signaling and *Csf1* upregulation. In this context, residual PDGFR signaling inhibits Wnt/β-catenin signaling. (**C**) During prolonged Scl-Ab exposure (6 weeks), a negative feedback mechanism, consisting of elevated expressions of the Wnt signaling inhibitor DKK1 and decreased expression of Wnt1 class of ligands, attenuates Wnt/β-catenin signaling, while Scl-Ab continues to prevent sclerostin-mediated coactivation of osteocatabolic PDGF-BB/PDGFR/ERK1/2 signaling.

Genetic ablation of PDGFR-encoding genes potentiated Scl-Ab–promoted trabecular bone mass gain. This effect was mainly due to an amplified early bone anabolic response to sclerostin neutralization (Figure 1). From a mechanistic point of view, deletion of PDGFR-encoding genes in preosteoblasts stimulated Wnt1-induced accumulation of active β-catenin and Wnt target gene expression (Figures 2 and 6), showing that PDGFRs negatively regulate the Wnt/β-catenin signaling pathway (Figure 7). The stimulation of bone formation by Scl-Ab therapy was only transient (Figure 1) (7–10, 14, 21), and the associated counterregulation of canonical Wnt signaling occurred in both control and *Pdgfr-cKO* mice (Figure 2), indicating that inhibitory action of PDGFRs on Wnt/β-catenin signaling in the presence of Scl-Ab is overridden by DKK1 and low Wnt1 class availability (Figure 7).

Scl-Ab treatment exerted sustained inhibition of bone resorption in control mice, as observed in humans (7–10). Scl-Ab administration transiently enhanced expression of *Wisp1*, a Wnt1-responsive negative regulator of osteoclastogenesis (22), and continuously reduced expression of *Csf1*, encoding an essential growth factor for osteoclastogenesis (Figure 3) (14). The differential regulation between *Wisp1* and *Csf1* expression by Scl-Ab suggested that sclerostin could regulate *Csf1* expression independently of Wnt/β-catenin signaling inhibition. Interestingly, Scl-Ab treatment could not further reduce *Csf1* expression in *Pdgfr-cKO* mice (Figure 3), thus indicating that PDGFR signaling is sufficient and necessary to explain sclerostin’s effects on *Csf1* expression and bone resorption. At the cellular level, short-term Scl-Ab treatment induced a slight additional decrease in osteoclast surfaces, while prolonged Scl-Ab exposure did not further reduce them in *Pdgfr-cKO* mice (Figure 3), thus reflecting the combination of a transient stimulatory effect on Wnt/β-catenin–mediated *Wisp1* and possibly *Opg* expression, and a continuous inhibitory effect on PDGFR-mediated *Csf1* expression (Figure 7). Altogether, those findings provide the cellular and molecular mechanisms involved in the biphasic inhibitory effects of Scl-Ab on bone resorption observed in clinical trials, i.e., a sharp decrease in serum CTX levels at 1 month, a return close to baseline at 3 months, followed by a progressive and continuous reduction at 6 and 12 months (7–9).

In line with the continuous inhibitory effect of Scl-Ab on PDGFR-mediated *Csf1* expression, the prolonged anticatabolic effect of Scl-Ab was associated with diminished expression of PDGFR target genes *c-Myc* and *Ccl2* in bone (Figure 4). The fact that Scl-Ab could prevent sclerostin-mediated potentiation of PDGF-BB–dependent PDGFR activation and *Csf1* expression in preosteoblast cultures and prevented calcitriol-induced osteoclast formation and activity in preosteoblast/osteoclast cocultures also supports our in vivo observations (Figure 5). Most importantly, we demonstrated that sclerostin, in contrast with DKK1, could function as a coactivator of PDGFRs and downstream ERK1/2 signaling pathways, independently of Wnt1 and Wnt/β-catenin signaling inhibitors (Figure 6), thereby explaining why bone resorption remains inhibited by prolonged Scl-Ab treatment despite the downregulation of bone formation (Figure 7). At a molecular level, heterodimerization between PDGFRs and LRP6 likely contributes to sclerostin’s effects on PDGFR signaling in preosteoblasts (Figures 5 and 7).

In conclusion, we identified what we believe is a new pathway for sclerostin’s effects on bone resorption and provide an explanation for the dissociation of Scl-Ab long-term therapeutic efficacy on bone resorption versus formation. Indeed, Scl-Ab’s anticatabolic effects on the skeleton occur by repressing PDGFR signaling and M-CSF expression in osteoblast lineage cells in a Wnt-independent manner. In addition, although it remains to be formally shown that activating mutations of PDGFRβ in osteoprogenitors contributes to osteopenia and occurrence of fractures in patients with Penttinen syndrome or Kosaki overgrowth syndrome (23), Scl-Ab could be useful to treat those pathological conditions. Finally, our findings also suggest that combination of PDGFR inhibition and sclerostin neutralization could represent a powerful approach to rapidly increase bone mass and strength in patients with osteolytic lesions provoked by multiple myeloma or bone metastases involving excessive PDGFR activity (24, 25).

## Methods

### Sex as a biological variable.

Our current study exclusively examined male mice. We expect our findings to be relevant to female mice as well, since PDGFR signaling in *Osterix*-positive cells exerts similar function (17) and Scl-Abs display similar effects on bone resorption and formation in both sexes (14, 21).

### Mice and Scl-Ab treatment.

*Pdgfr-cKO* mice in which *Pdgfra* and *Pdgfrb* genes can be selectively deleted under the control of an *Osterix* promoter following cessation of doxycycline treatment (Tet-Off system) were generated as previously described (17). Since both PDGFRs can compensate for the loss of the other and exert redundant function in osteoblast lineage cells (17), we used *Pdgfr-cKO* mice in our experiments. *Osx-Cre* mice were used as control animals. All mice were on a C57BL/6J genetic background. Sixteen-week-old male *Osx-Cre* and *Pdgfr-cKO* mice were randomly assigned to receive subcutaneous injections of 25 mg/kg Scl-Ab (r13c7, provided by UCB Pharma and Amgen Inc.) or an equivalent volume of saline solution twice a week for 2 weeks (bone formation rate peaked after 2 weeks of Scl-Ab treatment) or 6 weeks (bone formation rate returned to basal level after 6 weeks of Scl-Ab treatment) (Figure 1A) (21). *Cre* expression and consequent *Pdgfra* and *Pdgfrb* inactivation were induced 1 week prior to the onset of Scl-Ab treatments by stopping doxycycline administration (Figure 1A). Mice (3 to 6 animals per cage) were maintained under standard nonbarrier conditions, exposed to a 12-hour light/12-hour dark cycle, and had access to mouse diet RM3 containing 1.24 % calcium and 0.56 % available phosphorus (SDS Ltd) and water ad libitum. Experimental units were single animals. Mouse treatments (Veh or Scl-Ab) and endpoint measurements (by μCT and histomorphometry) were performed by different investigators. Investigators were blinded during endpoint measurements.

### Bone phenotyping.

Mice were sacrificed and their bones were excised for μCT analyses. Trabecular bone microarchitecture of proximal tibiae (100 slices from the beginning of secondary spongiosa) and cortical bone geometry of tibial midshafts (50 slices) were assessed using μCT (Viva-CT40, Scanco Medical) employing a 12-μm isotropic voxel size.

To measure dynamic indices of bone formation, mice received subcutaneous injections of calcein (10 mg/kg body weight; Sigma-Aldrich) at 9 and 2 days before euthanasia. Formalin-fixed undecalcified femurs were embedded in methylmethacrylate (Merck). Eight-micrometer transverse sections of midshafts and 8-μm sagittal sections of distal femurs were cut and mounted unstained for fluorescence visualization. Additional sagittal sections were stained with Goldner trichrome for osteoblast counting or with tartrate-resistant acid phosphatase (TRAP) substrate for osteoclast counting. Histomorphometric measurements were carried out using a Nikon Eclipse microscope and BioQuant software.

### Biochemistry.

Serum levels of PINP and TRAcP 5b were determined by using immunoassay kits (Immunodiagnostic Systems Ltd). M-CSF protein levels were determined in bone marrow supernatants (obtained by centrifugation at 1000*g* for 10 minutes) or in cell culture medium using Quantikine ELISA kits (R&D Systems). Results obtained with bone marrow supernatants were normalized to total protein levels.

### Osteoblast cultures.

Primary preosteoblasts were isolated from long bones of *Pdgfra^fl/fl^;Pdgfrb^fl/fl^* (*Pdgfr^fl/fl^*) mice as previously described (26). Briefly, bone chips were prepared from cleaned long bones and digested in 1 mg/mL collagenase II (Sigma-Aldrich) for 90 minutes at 37°C. Bone pieces were washed several times and incubated in α-MEM (Amimed, Bioconcept) containing 10% FBS (Gibco) for 9 days to allow cell migration from bone fragments. At that point, cells and bone chips were trypsinized (with trypsin/EDTA from Sigma-Aldrich) and passaged at a split ratio of 1:3. At the second passage, bone chips were removed. Medium was changed every 2–3 days. Preosteoblasts at passages 3–4 were used for in vitro experiments. *Pdgfr^fl/fl^* preosteoblasts were infected with 400 MOI of empty or Cre-expressing adenoviruses (Vector Biolabs) to obtain *Pdgfr^fl/fl^* (control) and *Pdgfr-cKO* preosteoblasts. Osteoblast differentiation was determined by incubating confluent preosteoblast cultures in osteogenic medium containing α-MEM, 10% FBS, 0.05 mM L-ascorbate-2-phosphate (Sigma-Aldrich), and 10 mM β-glycerophosphate (AppliChem GmbH) in the presence of Veh, 100 ng/mL Wnt1-sFRP1 (R&D Systems), with and without 500 ng/mL recombinant sclerostin (Peprotech).

### Cocultures.

For coculture experiments, primary *Pdgfr^fl/fl^* preosteoblasts infected with 400 MOI of empty or Cre-expressing adenoviruses were seeded at 30,000 cells per well in 24-well plates. The day after, nonadherent bone marrow cells isolated from WT mice were seeded over preosteoblasts at 300,000 cells per well in α-MEM supplemented with 10% FBS and treated with Veh, 10^–8^ M 1,25-dihydroxyvitamin D3 (calcitriol, Vit.D3), and/or 250 ng/mL recombinant murine sclerostin (Peprotech), with or without 1.25 μg/mL Scl-Ab or 500 ng/mL anti–M-CSF antibody (AF416, R&D Systems). After 8 days, cocultures were fixed and stained, and multinucleated TRAcP-positive cells were counted. To evaluate in vitro osteoclast-mediated demineralization, cocultures were performed under the same conditions in Corning Osteo Assay Surface multiple-well plates for 15 days. Multiple-well plates were cleaned with a bleaching solution and observed under an inverted phase-contrast microscope (Nikon Eclipse TE2000). Synthetic matrix demineralization was quantified using ImageJ software (NIH).

### Immunoprecipitations.

Confluent preosteoblast cultures were pretreated with Veh or 500 ng/mL recombinant sclerostin for 2 hours, and then treated with 25 ng/mL PDGF-BB for 15 minutes. Cell lysates were prepared by incubating osteoblast cultures in lysis buffer containing 1% NP-40 and phosphatase/protease inhibitors at 4°C for 30 minutes. Cells were then scraped and sonicated on ice for 10 seconds. Lysates were then centrifuged at 6000*g* for 30 minutes at 4°C. Cell lysates were incubated with 1:200 anti-LRP6 antibody (3395, Cell Signaling Technology [CST]) or isotype control overnight at 4°C. The day after, immunocomplexes were incubated with prewashed Protein G Magnetic beads (70024, CST) under agitation for 1 hour at room temperature. Then, beads were pelleted by using a magnetic separation rack and washed 3 times with cell lysis buffer. Immunocomplexes attached to beads were diluted with equal volumes of 2-fold–concentrated loading buffer and heated at 70°C for 30 minutes. Immunocomplexes were separated from magnetic beads by using a magnetic separation rack and collecting supernatants. Finally, supernatants were analyzed by Western blotting as described below.

### Western blots.

To measure PDGFR signaling, confluent preosteoblast cultures were pretreated with Veh or 500 ng/mL recombinant sclerostin with and without 2.5 μg/mL Scl-Ab for 1 hour, and then treated with 15 ng/mL PDGF-BB for 15 minutes. Preosteoblast cultures were rapidly frozen in liquid nitrogen and stored at –80°C until their use for analysis. Cell lysates were prepared by incubating cell cultures in RIPA buffer containing phosphatase and protease inhibitors at 4°C for 30 minutes. Lysates were then centrifuged at 6000*g* for 30 minutes. Lysate supernatants were diluted with equal volumes of 2-fold–concentrated reducing sample buffer. Those mixtures were then heated at 70°C for 30 minutes and subjected to gel electrophoresis in 6% to 15% gels. Proteins were electrotransferred to Immobilon P membranes (Merck Millipore) and immunoblotted with specific antibodies anti-PDGFRα (3174, CST), anti-PDGFRβ (3169, CST), anti–p-PDGFR (3170, CST), anti-LRP6 (3395, CST), anti–p-LRP6 (2568, CST), anti–p-STAT3 (9138, CST), anti–p-SHP2 (3751, CST), anti–p-ERK1/2 (9106, CST), anti-ERK1/2 (4695, CST), anti–p-β-catenin (Ser552) (9566, CST), anti–pan-actin (8456, CST), and anti–M-CSF (AF416, R&D Systems). Detection was performed using peroxidase-coupled secondary antibody, enhanced chemiluminescence reaction, and visualization by using G:Box gel analysis system (Syngene). Reprobed membranes were stripped according to the manufacturer’s protocol.

### RNA isolation and real-time PCR.

Total RNA was extracted from tibial metaphyses (whose bone marrow was removed by centrifugation at 16,200*g* for 20 seconds) or primary preosteoblast cultures using Tri Reagent (Molecular Research Center) and purified using an RNeasy Mini Kit (Qiagen). Single-stranded cDNA was synthesized from 2 μg of total RNA using a High-Capacity cDNA Archive Kit (Applied Biosystems) according to the manufacturer’s instructions. Real-time PCR was performed to measure the relative mRNA levels using the QuantStudio 5 Real-Time PCR System with SYBR Green Master Mix (Applied Biosystems). The primer sequences are provided in Supplemental Table 1. Melting curve analyses performed at the completion of PCR amplifications revealed a single dissociation peak for each primer pair. The mean mRNA levels were calculated from triplicate analyses of each sample. The obtained mRNA level for a gene of interest was normalized to β2-microglobulin mRNA level in the same sample.

### Statistics.

A sample size of 5 mice/group was required in order to detect a difference of 30% in fractional trabecular osteoclast surface (SD = 30%) between groups at the significance level of 0.01 and a power of 80%. In vitro experiments were performed in triplicate and independently repeated 3 or 4 times. Comparisons of multiple in vitro treatment groups were analyzed by 1-way ANOVA followed by Tukey’s multiple-comparison test. Interactions between effects of treatments and those of genotypes were analyzed by using 2-way ANOVA followed by Tukey’s multiple-comparison test. Interactions between effects of treatments, those of treatment durations, and those of genotypes, were analyzed by using 3-way ANOVA followed by Tukey’s multiple-comparison test. All quantitative data in Figures 1–4 are represented as box-and-whisker plots, with bounds from 25th to 75th percentile, median line, and whiskers ranging from minimum to maximum values.

### Study approval.

All performed experiments were in compliance with the guiding principles of the NIH *Guide for the Care and Use of Laboratory Animals* (National Academies Press, 2011) and approved by the Ethical Committee of the University of Geneva School of Medicine and the State of Geneva Veterinarian Office.

### Data availability.

All data supporting the findings of this study are reported in the Supporting Data Values file and available from Zenodo (https://zenodo.org) with the identifier doi:10.5281/zenodo.7670092.

## Author contributions

CT designed the study, developed methodology, acquired data, analyzed data, acquired funding, provided project administration and supervision, and wrote the original draft of and reviewed and edited the manuscript. PA and JB developed methodology, acquired data, and reviewed and edited the manuscript. JC reviewed and edited the manuscript. SF acquired funding and reviewed and edited the manuscript.

## Supplementary Material

Supplemental data

Unedited blot and gel images

Supporting data values

## Version 1 05/07/2024

In-Press Preview

## Version 2 05/22/2024

Electronic publication
