# Supplementary material for: Sclerostin blockade inhibits bone resorption through PDGF receptor signaling in osteoblast lineage cells
Source: JCI Insight. 2024 May 7;9(10):e176558. doi: 10.1172/jci.insight.176558 (PMC11141910; doi:10.1172/jci.insight.176558)

Unedited blots for Figure 5E

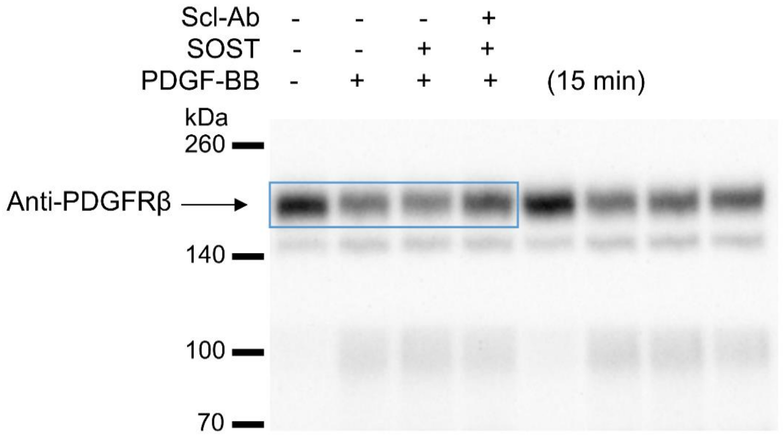

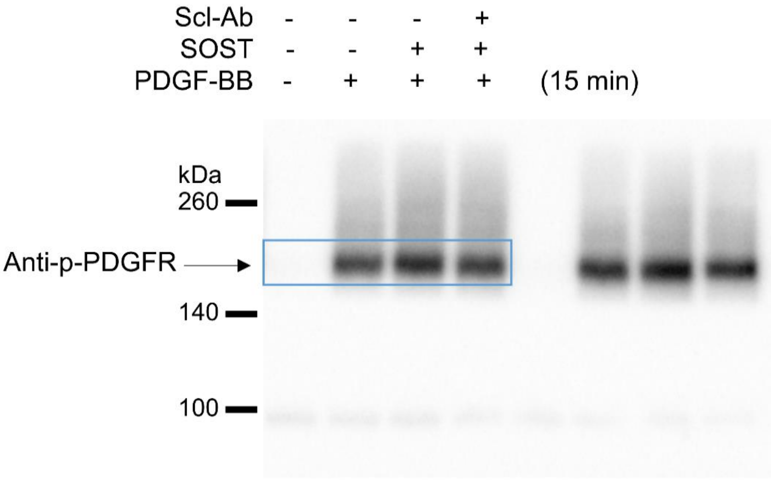

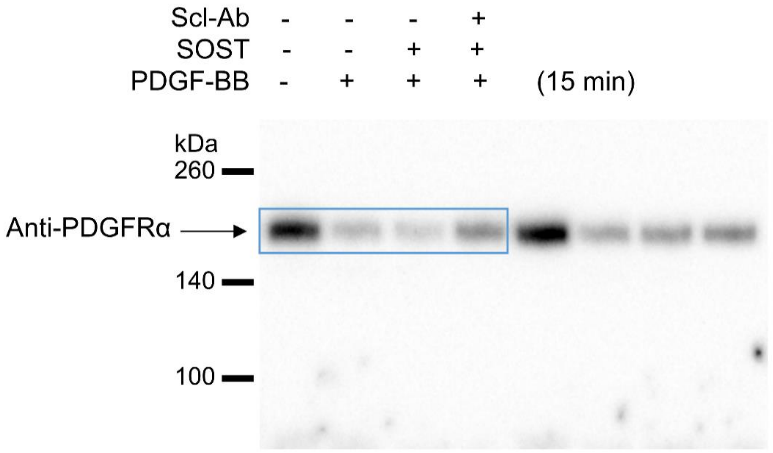

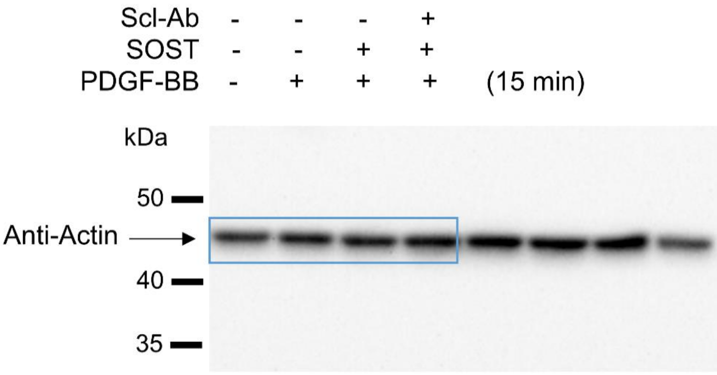

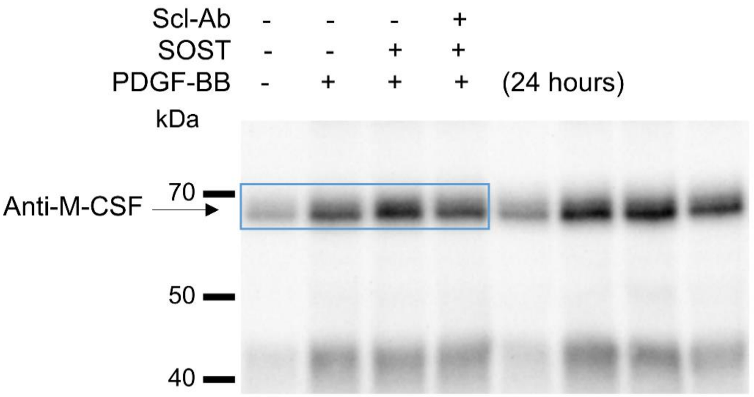

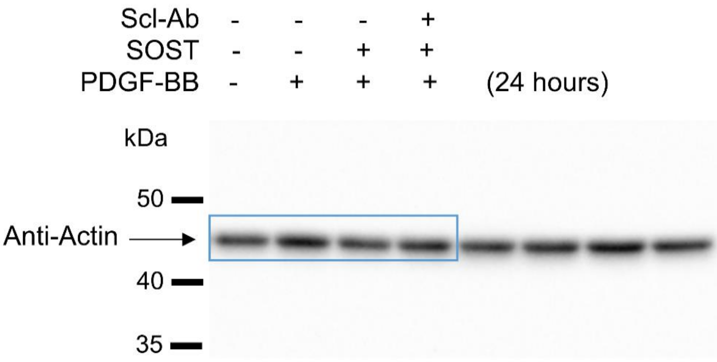

Unedited blots for Figure 5F

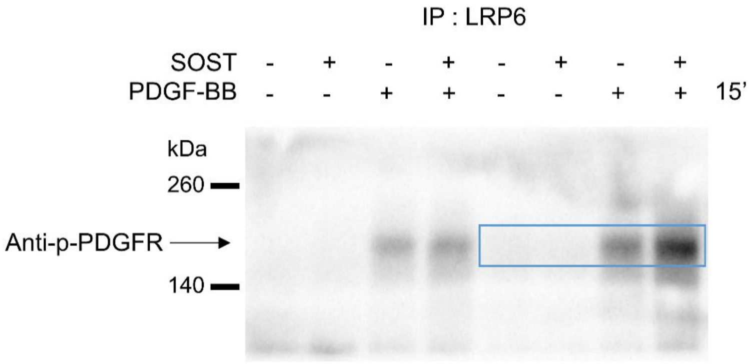

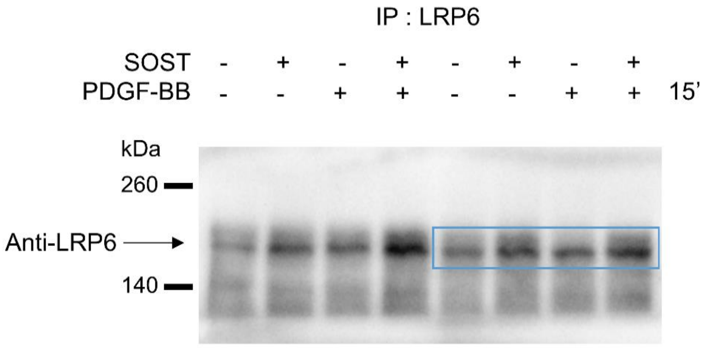

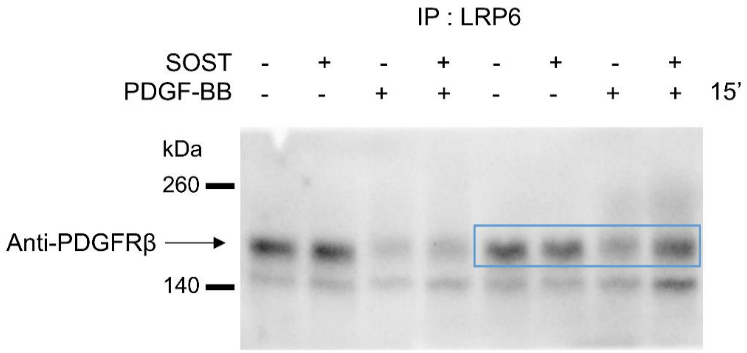

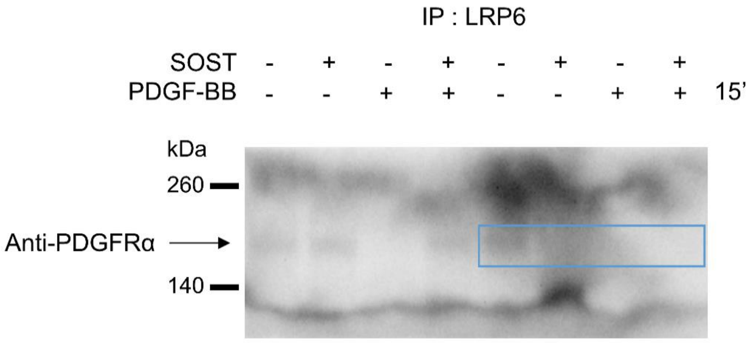

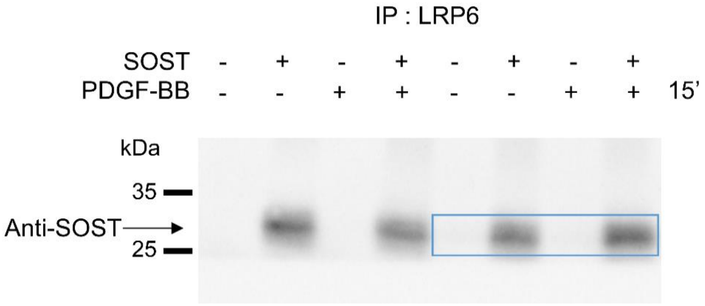

Unedited blots for Figure 5G

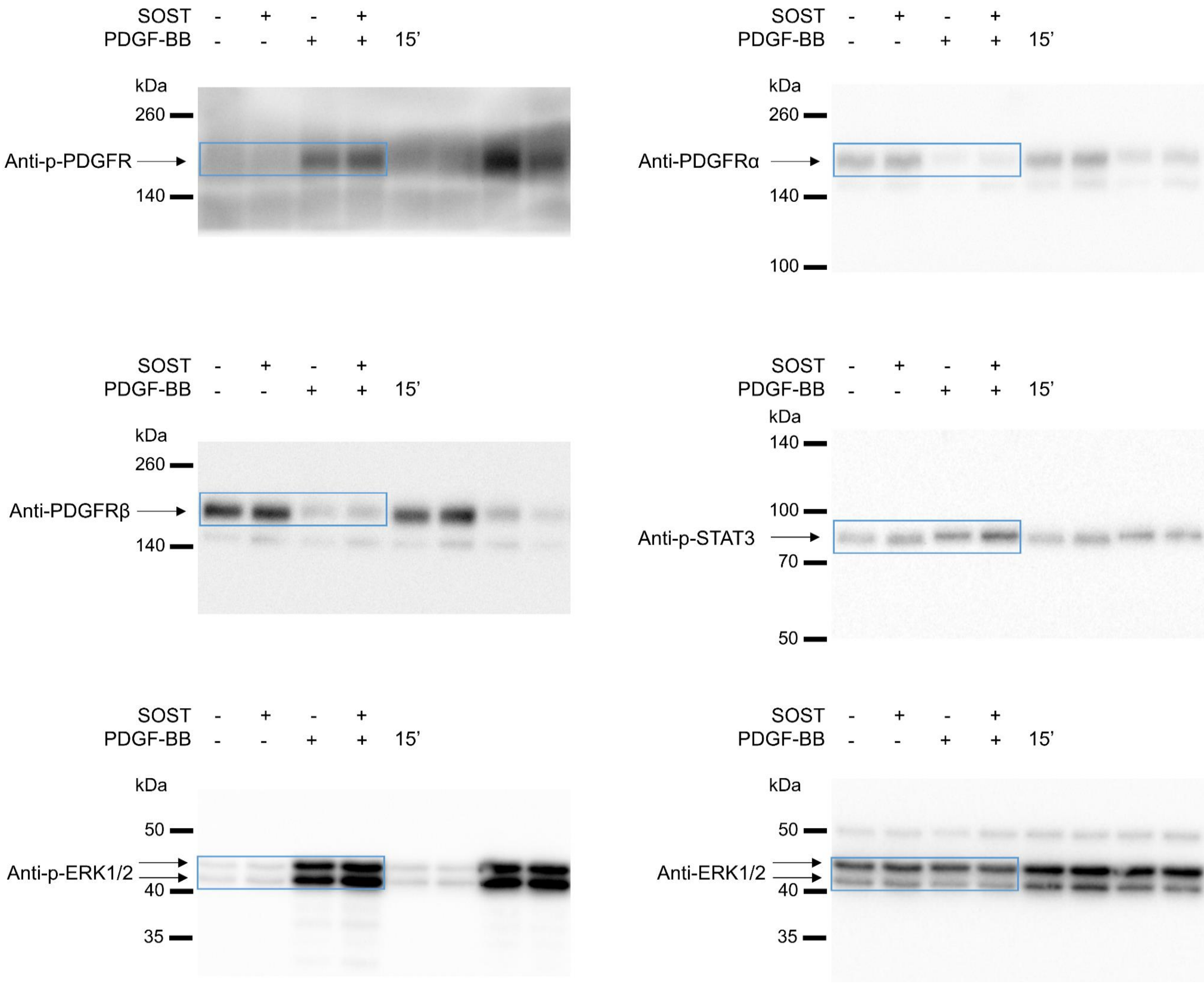

Unedited blots for Figure 6C

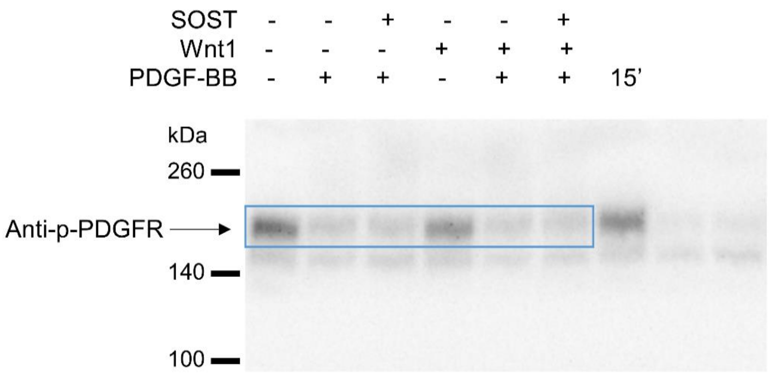

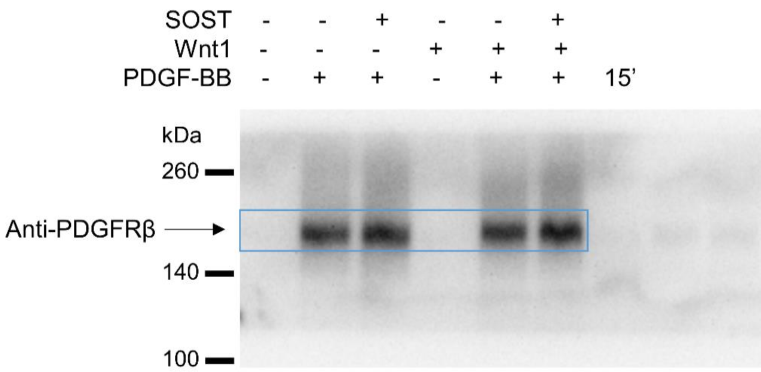

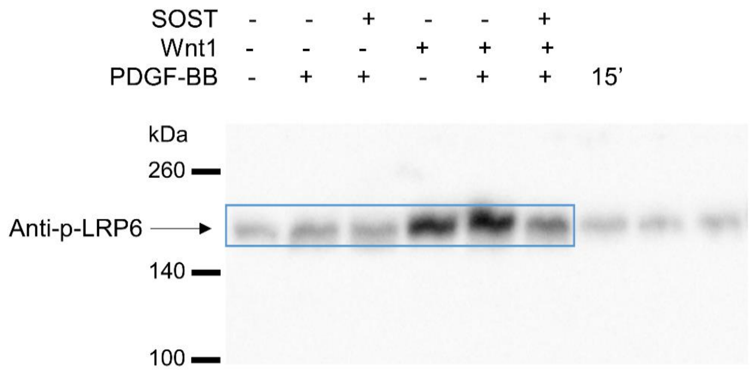

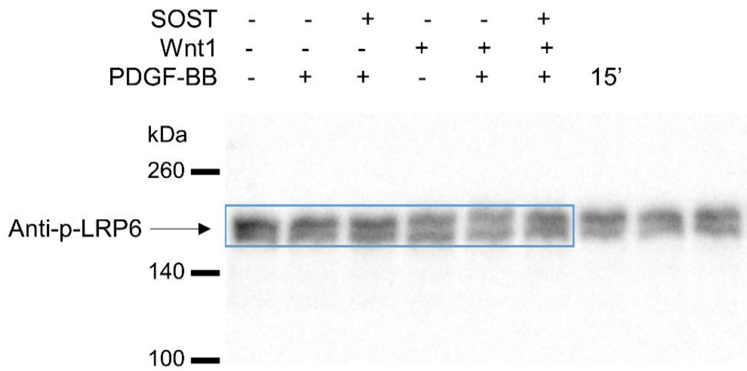

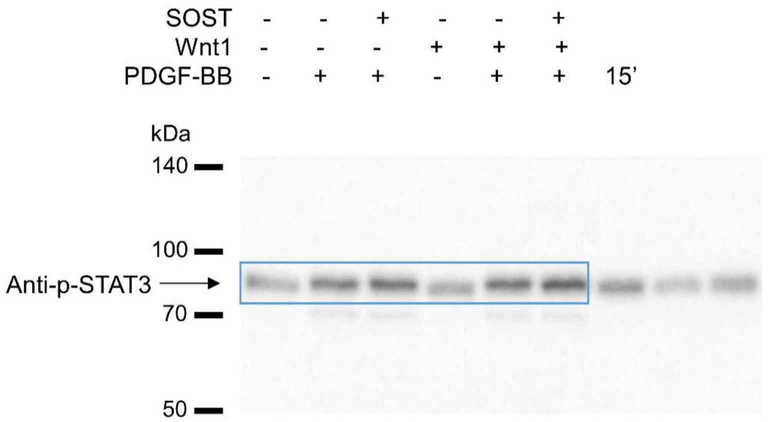

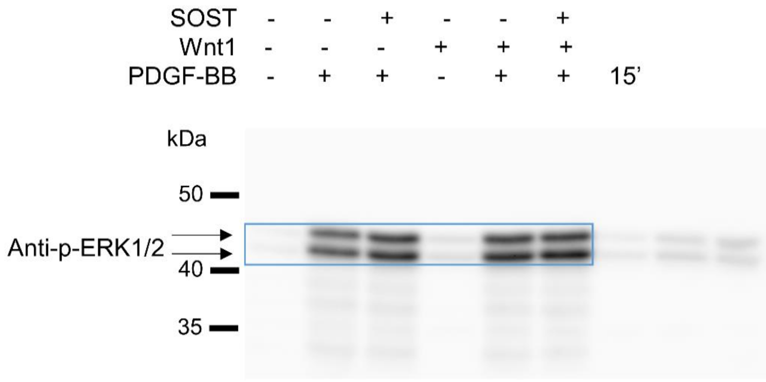

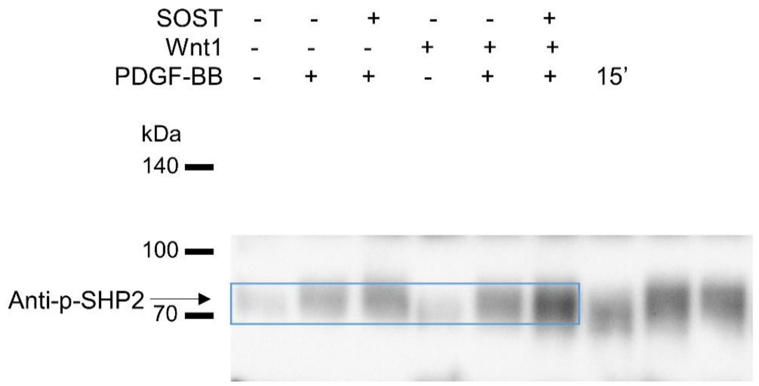

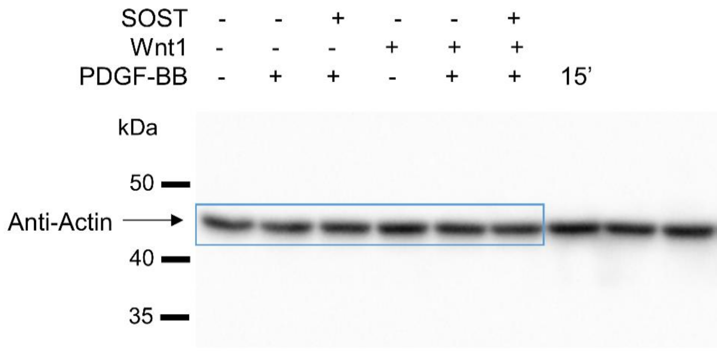

Unedited blots for Figure 6F

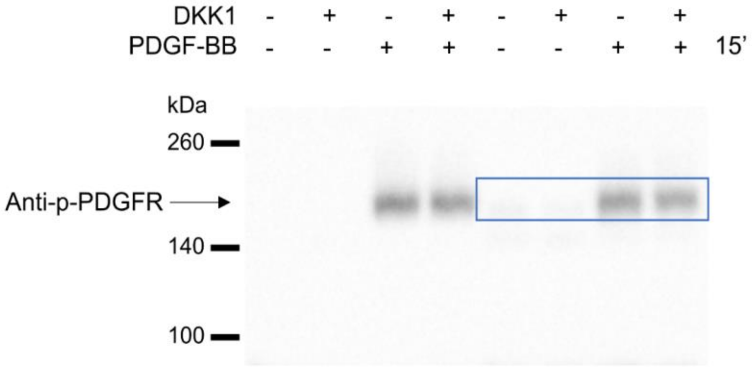

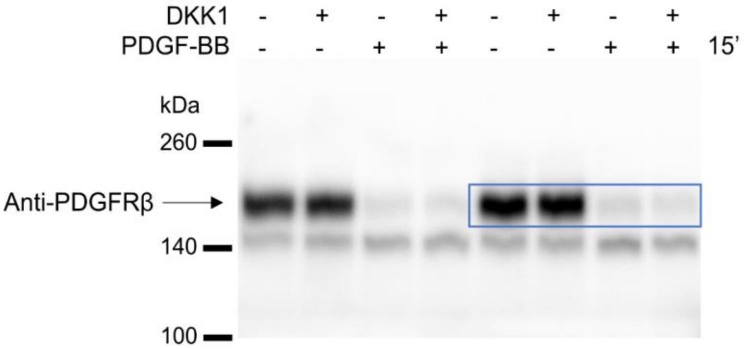

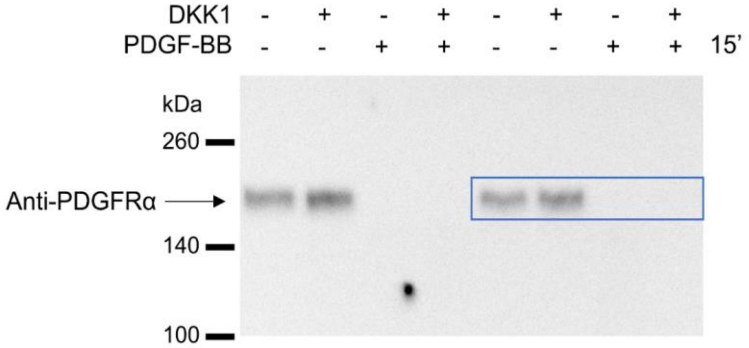

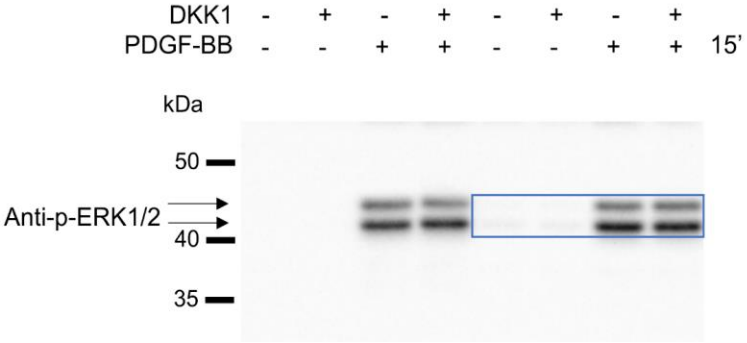

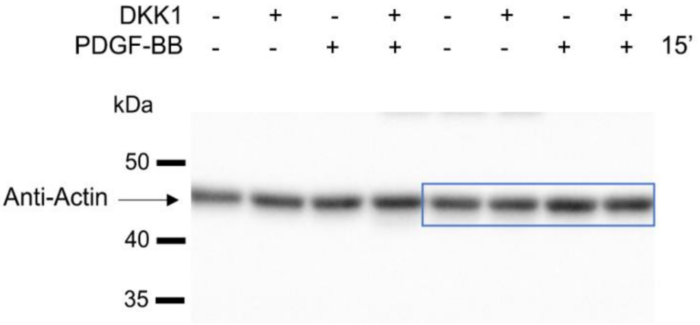

Unedited blots for Figure 6G

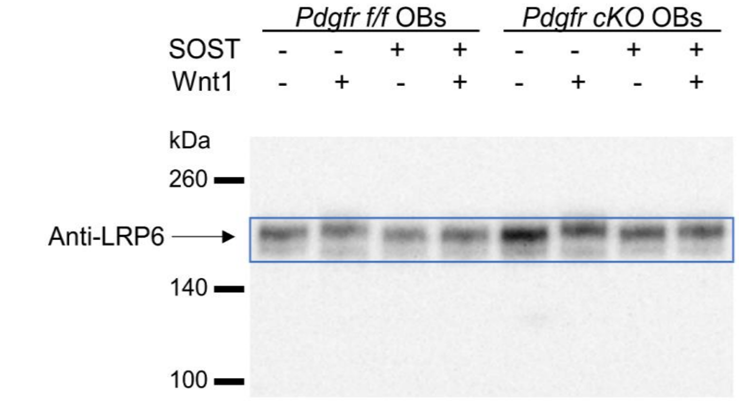

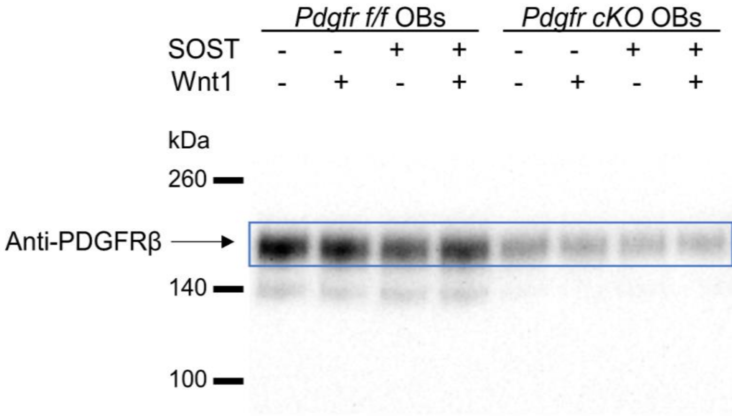

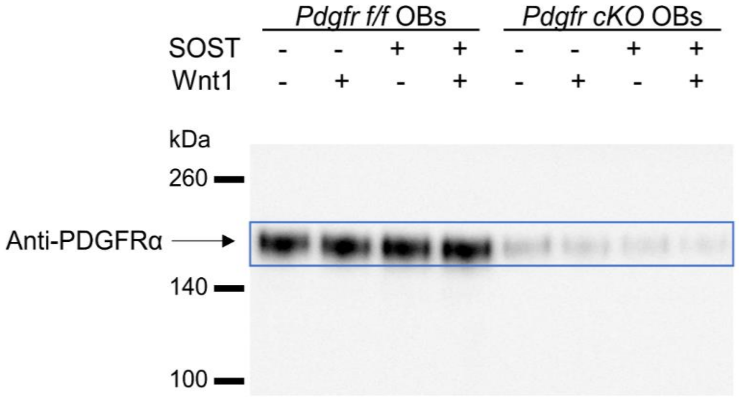

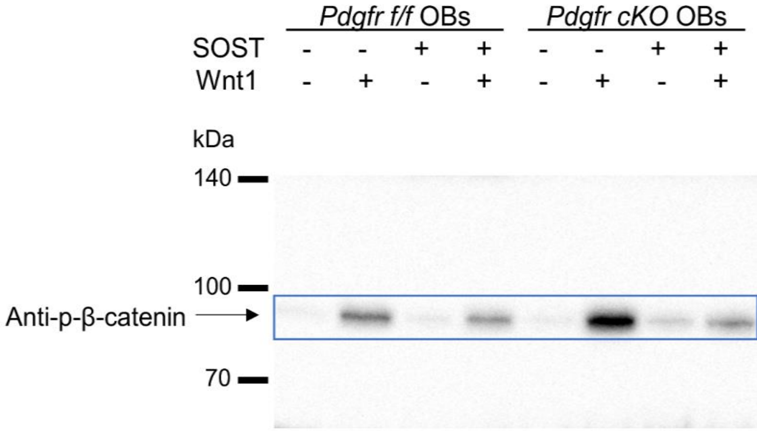

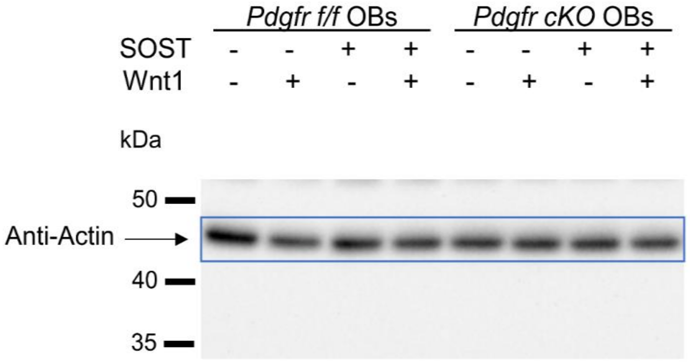

Unedited blots for Supplemental Figure 2C

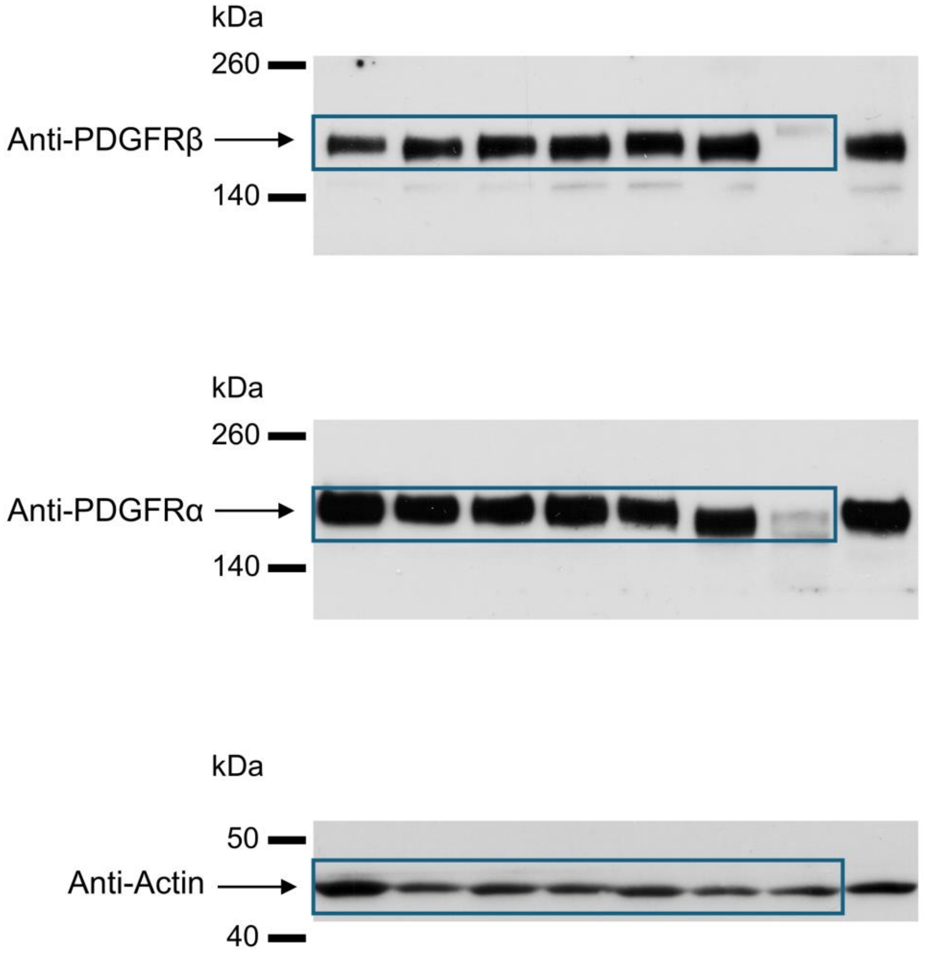

Supplement: Unedited blot and gel images [file jciinsight-9-176558-s124.pdf]
